# Whole-blood RNA transcript-based models can predict clinical response in two large independent clinical studies of patients with advanced melanoma treated with the checkpoint inhibitor, tremelimumab

**DOI:** 10.1186/s40425-017-0272-z

**Published:** 2017-08-15

**Authors:** Philip Friedlander, Karl Wassmann, Alan M. Christenfeld, David Fisher, Chrisann Kyi, John M. Kirkwood, Nina Bhardwaj, William K. Oh

**Affiliations:** 1 Division of Hematology and Medical Oncology, Tisch Cancer Institute, Icahn School of Medicine at Mount Sinai, Mount Sinai Hospital, New York, NY USA; 2 CPS Companion Diagnostics, Cambridge, MA USA; 3 Department of Dermatology, Harvard Medical School, Boston, MA USA; 4 Departments of Medicine, Dermatology and Translational Sciences, University of Pittsburgh School of Medicine, Pittsburgh, PA USA; 5 Parker Institute of Cancer Immunotherapy, San Francisco, CA USA

**Keywords:** Melanoma, Biomarker, Tremelimumab, CTLA-4, Predictive

## Abstract

**Background:**

Tremelimumab is an antibody that blocks CTLA-4 and demonstrates clinical efficacy in a subset of advanced melanoma patients. An unmet clinical need exists for blood-based response-predictive gene signatures to facilitate clinically effective and cost-efficient use of such immunotherapeutic interventions.

**Methods:**

Peripheral blood samples were collected in PAXgene® tubes from 210 treatment-naïve melanoma patients receiving tremelimumab in a worldwide, multicenter phase III study (discovery dataset). A central panel of radiologists determined objective response using RECIST criteria. Gene expression for 169 mRNA transcripts was measured using quantitative PCR. A 15-gene pre-treatment response-predictive classifier model was identified. An independent population (*N* = 150) of refractory melanoma patients receiving tremelimumab after chemotherapy enrolled in a worldwide phase II study (validation dataset). The classifier model, using the same genes, coefficients and constants for objective response and one-year survival after treatment, was applied to the validation dataset.

**Results:**

A 15-gene pre-treatment classifier model (containing ADAM17, CDK2, CDKN2A, DPP4, ERBB2, HLA-DRA, ICOS, ITGA4, LARGE, MYC, NAB2, NRAS, RHOC, TGFB1, and TIMP1) achieved an area under the curve (AUC) of 0.86 (95% confidence interval 0.81 to 0.91, *p* < 0.0001) for objective response and 0.6 (95% confidence interval 0.54 to 0.67, *p* = 0.0066) for one-year survival in the discovery set. This model was validated in the validation set with AUCs of 0.62 (95% confidence interval 0.54 to 0.70 *p* = 0.0455) for objective response and 0.68 for one-year survival (95% confidence interval 0.59 to 0.75 *p* = 0.0002).

**Conclusions:**

To our knowledge, this is the largest blood-based biomarker study of a checkpoint inhibitor, tremelimumab, which demonstrates a validated pre-treatment mRNA classifier model that predicts clinical response. The data suggest that the model captures a biological signature representative of genes needed for a robust anti-cancer immune response. It also identifies non-responders to tremelimumab at baseline prior to treatment.

**Electronic supplementary material:**

The online version of this article (doi:10.1186/s40425-017-0272-z) contains supplementary material, which is available to authorized users.

## Background

The value of immunotherapy in treating stage IV melanoma became indisputable in 2011, when the U.S. Food and Drug Administration approved ipilimumab, a CTLA-4 inhibitor. CTLA-4 expressed on the surface of activated T cells binds to B7 (CD80) on antigen-presenting cells with higher affinity than the co-stimulatory protein CD28. Disrupting B7’s binding to CD28 prevents T-cell co-stimulation, leading to dampening of the immune response. Ipilimumab is an IgG1 monoclonal antibody that binds CTLA-4 in an inhibitory manner. Treating relapsed or refractory stage IV melanoma with ipilimumab confers an overall survival benefit versus peptide vaccine treatment, with median survival increasing from 6 to 10 months and 2-year survival increasing from 14 to 24% [1]. The actual treatment response rate approximates 10% [1]. Benefit is durable, with the overall survival rate plateauing at 21% by year 3 and follow-up that now extends beyond year 10 [2].

Tremelimumab is an IgG2 monoclonal antibody that also inhibits CTLA-4. In a phase II trial in treatment-refractory metastatic melanoma, an objective response rate of 6.6% was observed [3]. A subsequent open label phase III trial in treatment-naïve patients demonstrated no survival benefit of tremelimumab compared with chemotherapy, although clinical efficacy was noted in a subset of patients (with a 10.7% objective response rate). Notably, the duration of response was significantly longer in responders to immunotherapy (35.8 vs. 13.7 months) [4]. Tremelimumab is currently undergoing further clinical investigation.

Disrupting other immune checkpoints, such as the interaction of programmed cell death protein-1 (PD-1) with its ligand PD-L1 can lead to clinical benefit. Melanoma cells express PD-L1, which binds to PD-1 on infiltrating T-cells, leading to decreased T-cell activity. Two PD-1 inhibitors, nivolumab and pembrolizumab, are FDA-approved for treating advanced melanoma, with response rates approximating 40% and 5-year survival post-nivolumab treatment of 35% [5–7]. Recently, concurrent CTLA-4 and PD-1 inhibition (ipilimumab plus nivolumab) was FDA-approved on the basis of a 57.6% response rate [8].

The development of antibodies which inhibit CTLA-4 and PD-1 have improved the efficacy of treatment available to patients with stage IV melanoma. While treatment benefits a subset of patients, many do not respond. Identifying biomarkers which reliably identify treatment responders and long term survivors would allow for early selection of alternative treatment in patients unlikely to benefit, while limiting toxicity risk and health care expenditures. Of patients treated with ipilimumab monotherapy, PD-1 inhibitor monotherapy, or ipilimumab plus nivolumab combination therapy, 27%, 17% and 55%, respectively, develop high-grade toxicities [8]. The annual regimen price per patient in the US is approximately $120,000 for ipilimumab and $150,000 for either of the two PD-1 inhibitors. The combination of ipilimumab plus nivolumab costs approximately $256,000 per patient. The Centers for Medicare & Medicaid Services has reimbursed most of these immunotherapy costs over the past two years, however, European reimbursement rates are far lower: France, the UK, Germany and Italy reimburse approximately 55%, 40%, 30%, and 30% of the cost, respectively [9]. Development of a pretreatment biomarker predictive of efficacy to anti-CTLA4 or anti-PD1 blockade would allow for selection of patients more likely to respond to treatment and guide efficacious treatment selection. Expensive immunotherapies could be selectively recommended to patients likely to respond. This would not only prevent patients unlikely to respond from developing toxicity to ineffective therapy but also could lower cost to the health care system through decreased spending on expensive but ineffective therapy.

Discovery of biomarkers robustly predictive of treatment efficacy prior to actually initiating treatment should optimize treatment planning, as well as limit toxicity risk and healthcare expenditure. Still, no tissue- or blood-based biomarker is approved to select melanoma patients for immunotherapy. While a tissue-based biomarker, PD-L1 IHC 28–8 pharmDx (Dako), is commercially available, data do not support its ability to determine prior to treatment which patients will respond to anti-PD-1 immunotherapy [10]. Melanoma patients with low PD-L1 (<5%) tumor expression have 41.3 and 54.8% response rates to nivolumab and combined nivolumab plus ipilimumab treatment, respectively [11].

We reexamined data of 169 mRNA-based transcripts from whole blood collected prior to treatment with tremelimumab in melanoma patients in two large, independent clinical trials. Previously, we reported a whole blood 4-gene mRNA signature was predictive for overall survival in melanoma patients treated with tremelimumab. We demonstrated that CTSD, PLA2G7, TXNRD1, and IRAK3 expression levels in peripheral blood predict overall survival [12]. However this signature did not predict for response to tremelimumab.

The current analysis uses a novel computational systems biology approach, exploiting more recent knowledge of the mechanism of checkpoint inhibition. We proceeded with the hypothesis that whole blood RNA transcript-based genes predictive for both objective response and survival could be identified pre-treatment in advanced melanoma patients treated with tremelimumab. We define a pre-treatment mRNA gene signature obtained from blood that predicts response.

## Methods

### Patient population

The patient population in Table 1 has been previously described [12]. Both the discovery and validation datasets resulted from pre-treatment blood samples collected in multinational, open-label studies of tremelimumab administered to advanced melanoma patients. Only patients from whom both a pre- and a post-treatment blood sample were available were included in our analysis. The pre-treatment discovery dataset was a randomized phase III study which treated 325 patients with tremelimumab of which 210 patients had both a pre- and a post-treatment blood sample available with *N* = 28 responders and *N* = 182 non-responders [4]. The pre-treatment validation dataset was a non-randomized phase IIb study which enrolled 251 patients of which 150 patients had both a pre- and a post-treatment blood sample available with *N* = 20 responders and *N* = 130 non-responders [3]. The phase IIb study initially reported responses in 16 patients but subsequent central radiology review determined that responses developed in 20 patients. Response results for the phase III study were also determined by central radiologist review of imaging. For our analysis response determination was based upon the results of the central radiology reviews. In both studies, response was determined by RECIST criteria [13]. The patients in the pre-treatment discovery dataset were treatment-naïve, while the patients in the pre-treatment validation dataset were chemotherapy-refractory. Therefore, the pre-treatment discovery dataset patients had a longer median survival of 13 months compared with the validation dataset patients at 8.8 months. Similarly, one-year survival for the discovery and validation datasets was 56 and 29%, respectively.

**Table 1:** Demographics of patients in the discovery and validation populations

	Discovery data set	Validation data set
Number of patients	210	150
Age, median (range) years	59 (22–90)	53 (18–89)
Gender n (%)		
Male	117 (56%)	94 (63%)
Female	93 (44%)	56 (37%)
Objective Response n (%)		
Patient responsive	28 (13%)	20 (13%)
Patients non-responsive	182 (87%)	130 (87%)
One-year Survival n (%)		
Patient alive at one year	118 (56%)	43 (29%)
Patient deceased at one year	92 (44%)	107 (71%)
Prior chemotherapy	No	Yes
Stage of disease n (%)		
IIIC	13 (6%)	5 (3%)
IV M1A	35 (17%)	16 (11%)
IV M1B	49 (23%)	30 (20%)
IV M1C	113 (54%)	99 (66%)
Live in United States n (%)		
U.S.	44 (21%)	62 (41%)
Non-U.S.	166 (79%)	88 (59%)

### Gene selection and sample processing

The selection process for the 169 genes tested has been described previously and included genes associated with inflammation, immunity, the CTLA4 pathway, oncogenes, or found to discriminate melanoma versus normal in previous exploratory studies [12]. Genes that discriminate between melanomas and healthy normals were determined by transcriptome profiling with microarray analysis identifying 78 such genes from which quantitative PCR confirmed the 27 melanoma associated genes included in the 169 gene panel [12, 14]. Additional file 1 lists the full gene names and aliases.

Sample processing procedures were as described [12]. Whole-blood was collected in PAXgene® tubes and processed to RNA that met quality and integrity standards (RNA integrity number ≥ 6.3) per the Bioanalyzer 2100 in combination with the RNA 6000 Nano or Pico Series II LabChip. First-strand complementary DNA was synthesized from random hexamer-primed RNA templates using TaqMan® reverse-transcription reagents. Individual target-gene amplification was multiplexed with the 18S rRNA endogenous control and run in triplicate in 384-well format on the 7900HT fast real-time PCR system.

### Statistical analysis

We tested the hypothesis in the discovery dataset and validated it in the independent validation dataset. Genes in both datasets are highly correlated and contain both predictors and “enhancer” variables, as first defined by Horst [15]. An enhancer variable, while not itself predictive of the outcome, is highly correlated with individual genes that are predictive of the criterion. Given technical variability, we eliminated low-expressing genes. With each heating/cooling cycle, the real time PCR assay detects fluorescent signal accumulation. The Ct (cycle threshold) is the number of cycles required for the fluorescent signal to exceed background level. Thermal cycling goes through 40 cycles, and 18S rRNA is measured at the 14th cycle. Delta Ct is measured 26 cycles from the point endogenous control is recognized.

As part of our Quality Control program, mRNA transcript sets were assessed for technical variations using different reagent batches, operators and instruments. Technical variation for genes with delta Ct of ≤20 was under 0.25 and increased to 0.5 when delta Ct was 20 to 25. Delta Ct of 23 was selected as a weak-reactions cutoff.

All pre-treatment genes in the 169-gene panel were assessed by the analysis of variance (ANOVA) t-test and the Mann-Whitney U Test for Unknown Distributions to determine predicators with a *p* value <0.05. Both ANOVA t and Mann-Whitney U test results were determined for all individual predictor genes. Candidate synergistic 2-gene pairs included either predictors or a predicator plus an enhancer variable.

We used Statistical Innovations’ CORExpress 1.1 commercial software, which employs correlated component regression analysis and handles multicolinearity due to correlated predictors effectively even with high-dimensional data. The software was run in two-component mode for synergistic gene pair analysis. The final list of synergistic gene pairs, or “2-gene core models,” was trained to predict response in the discovery dataset, tested next in the validation dataset based on objective response and then tested using one-year survival as the criterion. Over 260 pre-treatment response-predictive 2-gene core models were validated for objective response and survival.

Larger, optimal pre-treatment classifier models were constructed by combining validated 2-gene core models using CORExpress’ correlated component regression package. The software was run in three-component, step-down mode starting with validated pre-treatment core models to eliminate weaker genes. At each step, resulting classifier models were validated for response and survival on the validation dataset. The discovery dataset’s AUC was checked with publically-available MedCalc version 17 ROC analysis and *p*-value software [16]. The use of the CORExpress® software handles multi-testing, also called high-dimensional data, in which the number of potential predictors exceeds the number of test samples. “CORExpress® 1.1 implements Correlated Component Regression (CCR) and focuses on regression analysis with a large numbers of correlated predictors *P* which may exceed the sample size *n*” Additional details can be obtained at the following website: http://www.statisticalinnovations.com/shop/corexpress/.

After development of a 15-gene predictive model for response using the discovery pre-treatment dataset and this methodology, the 15-gene algorithm was tested for both prediction of response and survival using the pre-treatment validation dataset. Subsequently, the 15-gene pre-treatment algorithm was tested for response and 1-year survival using both the discovery and validation post-treatment datasets. One year survival was assessed as 1-year survival outcome data was available and provides early timepoint to assess the ability of the gene signature to discriminate for survival outcome but may not fully reflect multi-year survivial as with another CTLA-4 inhibitor, ipilimumab, longer term survival plateaus at approximately 3-years. The step-wise statistical analysis utilized is outlined in Table 2.

**Table 2:** Algorithm of the stepwise statistical analysis perfomed on the discovery and validation datasets

Step-Wise Statistical Analysis Using Discovery/Validation Methodology
Step 1: 2-Gene Models to Predict Immunotherapy Response and Survival
CORExpress 1.1 regression analysis software for high-dimensional data
Train 2-gene models with pre-treatment Discovery dataset *N* = 210
Test 2-gene models with pre-treatment Validation dataset *N* = 150
Over 260 2-gene synergistic pre-treatment models trained and validated
Step 2: Larger Gene Models to More Accurately Predict Response and Survival
Include only genes validated in 2-gene models from Step 1
Optimize model coefficients using CORExpress 1.1 software
Train optimized models with pre-treatment Discovery dataset *N* = 210
Test optimized models with pre-treatment Validation dataset *N* = 150
Step 3: Finalize 15-Gene Model to Predict Response and Survival
Optimal 15-gene pre-treatment model selected from Step 3
Use MedCalc version 17 software for ROC and *p*-value analysis
Test 15-gene model with pre-treatment Discovery dataset *N* = 210
Test 15-gene model with pre-treatment validation dataset *N* = 150
Step 4: Test Pre-treatment 15-Gene Model with Post-Treatment Datasets
15-gene pre-treatment response and survival model from Step 3
Use MedCalc version 17 software for ROC and *p*-value analysis
Test 15-gene model with post-treatment Discovery dataset *N* = 210
Test 15-gene model with post-treatment Validation dataset *N* = 150

The gene expression of components of the pretreatment gene signature was also compared in discovery dataset responders and nonresponders to expression in a set of 50 blood bank healthy normal volunteers. The Healthy Normal volunteers were *N* = 25 female and *N* = 25 male [14].

## Results

**Fig. 1:**
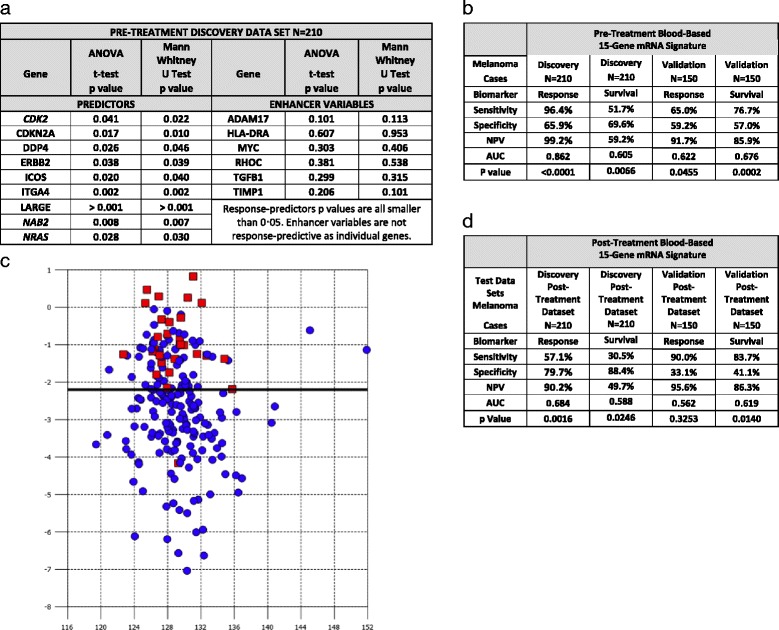
The 15-gene signature predicting response in the pre-treatment discovery data set consists of 9 predictor genes and 6 enhancer variable genes (**a**). The sensitivity, specificity, negative predictive value, area under the curve and p-value of the pre-treatment 15 gene signature predicting response in the discovery data set and both response and survival in the validation dataset (**b**). Composite response-prediction score generated to visually represent responders versus non responders. X Axis is the correlated component score and Y axis is the composite response-prediction score. Red squares (responders) and blue circles (non-responders) (**c**). Testing of the post-treatment discovery and validation datasets prediction of response and survival including the sensitivity, specificity, negative predictive value, area under the curve and p-value using the pre-treatment 15 gene signature (**d**)

Our goal was to identify peripheral blood-based biomarkers that predict response and one year survival to tremelimumab using blood samples collected before treatment initiation. Samples were available for 210 discovery dataset patients. The phase III trial was chosen as the discovery dataset because it required patients to be treatment-naïve, whereas patients in the phase II trial had received prior chemotherapy. Response was assessed 10 months after tremelimumab initiation. The treatment responses seen in both studies were mostly partial and in our analysis patients were identified as being a responder or a nonresponder. Using correlated component regression analysis we identified a 15-gene pre-treatment classifier model as optimal in terms of AUC for the discovery dataset. The 15-gene pre-treatment model consists of 9 predictors and six non-predictive enhancer variables, as illustrated in Fig. 1a.

The 15-gene pre-treatment model is represented by the formula:

Predicted pre-treatment to respond to tremelimumab >24.009-(0.8697xADAM17) + (0.7486xCDK2)-(0.5885xCDKN2A) + (0.3462xDPP4)-(0.2401xERBB2) + (1.7427xHLA-DRA) + (0.2481xICOS)-(1.1975xITGA4)-(1.0184xLARGE) + (1.1721xMYC)-(0.6531xNAB2)-(1.1491xNRAS) + (0.7377xRHOC)-(1.0585xTGFB1) + (0.8328xTIMP1).

As Fig. 1b illustrates the model predicted response with a negative predictive value (NPV), z statistic and AUC, of 99.2%, 10.1, and 0.862 (95% confidence interval 0.807 to 0.905, *p* < 0.0001), respectively. The model predicted one-year survival with a NPV, z statistic and AUC, of 59.2%, 2.72, and 0.605 (95% confidence interval 0.536 to 0.672, *p* = 0.0066), respectively. The model was applied subsequently to the independent validation dataset of 150 melanoma patients. The genes, constant and gene coefficients remained the same as in the discovery model and a fixed cutoff was used. The validation dataset NPV, z statistic, AUC were 91.7%, 2.0, and 0.622 (95% confidence interval 0.539 to 0.699, *p* = 0·0455), respectively, thereby validating the model’s ability to predict response (Table 2b). The pre-treatment model was also predictive of one–year survival with a NPV of 85.9%, z statistic of 3.7, and AUC of 0.676 (95% confidence interval 0.594 to 0.750, *p* = 0.0002). The composite pre-treatment response prediction scores demonstrated clear differentiation between responders and non-responders (Fig. 1c).

As Fig. 1d illustrates the 15-gene pre-treatment model also predicted response using the discovery post-treatment dataset with a NPV, z statistic and AUC, of 92.4%, 3.2, and 0.684 (95% confidence interval 0.616 to 0.746, *p* = 0.0016), respectively. The model also predicted survival in the post-treatment dataset with a NPV, z statistic and AUC, of 49.7%, 2.25, and 0.588 (95% confidence interval 0.518 to 0.655 *p* = 0.0246), respectively. The model did not predict for response using the validation post-treatment dataset with a NPV, z statistic and AUC, of 95.6%, 0.98, and 0.592 (95% confidence interval 0.478 to 0.642, and *p* = 0.3253), respectively. The model, however, predicted survival in the validation post-treatment dataset with a NPV, z statistic and AUC, of 86.3%, 2.6, and 0.619 (95% confidence interval 0.536 to 0.697, and *p* = 0.0140), respectively.

While the 15-gene signature predicted for response and one year survival, many other genes were up-regulated in a statistically significant manner in responders versus non-responders in the phase III trial. However, these same genes showed no difference in responders versus non-responders in the validation phase II trial. CTLA4, for example, showed a statistically significant pre-treatment response-predictive ANOVA t-test (*p* value = 0.005) in the phase III but not the phase II where the (*p* value = 0.346.) Table 3 shows eight examples of phase III pre-treatment response-predictors that are not statistically significant in the phase II study.

**Table 3:** Examples of genes predictive for response in the discovery but not validation datasets

Gene	*N* = 210 Discovery Pre-treatment ANOVA t-test	*N* = 150 Validation Pre-treatment ANOVA t-test
CD28	0.026	0.158
CD80	0.012	0.368
FAIM3	0.008	0.638
FYN	0.006	0.962
IL18BP	0.020	0.958
IL32	0.021	0.686
IL7R	0.009	0.590
INPP4B	0.006	0.740

The data highly suggest that the expression of the 15-genes in the signature represent expression levels of particular genes needed for robust immune responses against cancer. Expression of these genes may identify patients whose immune systems are already primed to have an anti-cancer immune response. Therefore the level at which the 15 genes were expressed in discovery dataset responders was compared to expression in a set of 50 blood bank healthy normal volunteers. Unexpectedly only 6 genes demonstrated differential expression being either up or down regulated when responders were compared to healthy normal controls of which 5 of these 6 genes were enhancer genes. Eight of the genes demonstrated equivalent expression between responders and healthy normal controls (Table 4) of which 7 of these 8 genes were predictor genes.

**Table 4:** Relative gene expression of the 15 genes comprising the pre-treatment signature comparing responders in the discovery dataset to healthy volunteers and to non-responders

15-Gene Pre-Treatment Model	Predictor or Enhancer Variable	Blood Bank	Difference Normals versus Responders	Phase 3 Discovery Dataset		
		*N* = 50		*N* = 28	*N* = 182	Difference
		Healthy Normals		Responders	Non-Responders	Responders vs Non-responders
Responders Equivalent to Normals						
ITGA4	Predictor	14.2	0.02	14.22	14.61	0.39
LARGE	Predictor	22.0	0.09	22.09	22.97	0.88
CDK2	Predictor	19.6	0.09	19.69	19.91	0.22
TIMP1	Enhancer	15.0	0.10	15.1	14.95	−0.15
DPP4	Predictor	18.5	0.12	18.62	18.95	0.33
NRAS	Predictor	17.1	0.13	17.23	17.44	0.21
ERBB2	Predictor	23.0	−0.18	22.82	23.23	0.41
NAB2	Predictor	20.0	−0.29	19.71	20.04	0.33
Responders Upregulated Compared to Normals						
ADAM17	Enhancer	18.5	0.32	18.18	18.36	0.18
RHOC	Enhancer	16.9	0.39	16.51	16.63	0.12
TGFB1	Enhancer	13.4	0.45	12.95	13.05	0.10
CDKN2A	Predictor	21.4	0.63	20.77	21.16	0.39
Responders Downregulated Compared to Normals						
HLADRA	Enhancer	12.1	0.48	12.58	12.64	0.06
MYC	Enhancer	17.7	0.82	18.53	18.67	0.14
Measurement of Gene Expression Not Available						
ICOS	Predictor	N/A	N/A	22.32	22.78	0.46

As discussed above, the 15-gene signature contains 6 enhancer variable genes which do not independently predict response but rather enhance the predictive ability of the 9 predictor genes. While gene expression of most of the enhancers differed between responders and healthy normals only 1 (potentially 2) of the 9 predictive genes demonstrated differential expression (ICOS is a predictive gene but its mRNA expression was not available for measurement in the healthy normals).

Given that almost all of the predictor genes showed equivalent expression in responders and healthy normals we hypothesized that the predictor genes were differentially expressed in the non-responders. As shown in Table 4 all eight evaluable predictive genes were in fact down-regulated in the non-responders relative to responders. However all six enhancers showed no significant change in gene expression between responders and non-responders.

## Discussion

An ideal biomarker should be obtained easily with minimal risk to the patient. Biomarkers based on mRNA transcript gene expression profiling obtained from whole blood have enormous advantages over tumor-based gene expression profiling. Tumor biopsies are invasive and difficult to obtain. Blood samples are much less invasive and less costly to obtain, have minimal risk, can be serially obtained, and are collectible by non-physicians. Potential biomarkers of efficacy following CTLA-4 blockade have been reported based on characteristics present in peripheral blood, including T-lymphocyte ICOS expression, neutrophil/lymphocyte ratio, CTLA-4 polymorphisms, effector/suppressor T-lymphocyte ratio, absolute lymphocyte and eosinophil counts, T-cell receptor diversity, and nomogram model score comprising baseline lactate dehydrogenase value and absolute neutrophil count [17]. These markers need validation.

The pretreatment gene signature we identified and validated contains 9 predictor genes. The remaining 6 genes are enhancer variable genes which do not individually predict for response but rather enhance the predictive ability of the predictors. Use of enhancer variables decreases extraneous variation and strengthens the relationship between a predictor and a given criterion (response rate or survival). Understanding the function of the predictive genes can help identify cellular processes associated with benefit following inhibition of CTLA-4 activity.

Given that tremelimumab is immunomodulatory, one might hypothesize that a gene expression signature predicting efficacy would encompass predominately genes directly modulating immune activity or the tremelimumab target CTLA-4. However only two of the nine predictor genes (ICOS and DPP4) are direct modulators of immune activity. ICOS is an inducible co-stimulatory molecule expressed on active CD4+ T-lymphocytes [18]. DPP4, a dipeptidyl peptidase subfamily member of serine proteases, regulates chemokine activity [19]. Baseline gene expression of the tremelimumab target CTLA-4 is not a component of the signature and not a validated predictive gene.

However the immune milieu of the tumor microenvironment could be modulated through complex mechanisms which facilitate responsiveness to CTLA-4 inhibition. Activation of signal transduction pathways such as the MAPK pathway and changes to extracellular components can alter the immune activity in tumor microenvironments. Five of the predictor genes are components of signal transduction pathways or regulate cell cycle progression. The genes associated with signal transduction affect multiple pathways but the MAPK and PI3K/AKT pathways in particular. NRAS regulates signal transduction pathway activity, including MAPK and AKT pathways. ERBB2 dimerizes with EGFR, modulating downstream ERK and AKT signaling [20] NAB2 regulates mRNA transcripts targeting for export through the nuclear pore [21].

The genes directly associated with cell cycle progression regulate different phases of the cycle. CDKN2A encodes p16, a tumor suppressor that binds CDK4/6 inhibiting cyclin-dependent kinase activity regulating the G1 portion of the cycle [22]. CDK2 is a cyclin-dependent kinase that regulates mitotic entry [23]. Two other predictor genes regulate extracellular matrix integrity (LARGE, a glycosyltransferase with substrates involved in cellular connection to basement membranes [24] and ITGA4, an integrin regulating cell adhesion to the extracellular matrix). Therefore, the combined mRNA expression of key signal transduction pathway components and regulators of the extracellular tumor microenvironment in combination with certain direct regulators of immune activity (ICOS, DPP4) appears to predict priming for responsiveness to CTLA-4 blockade.

We found that relative to healthy normal controls expression of the response predictive genes was preserved in responders but significantly down regulated in non-responders. The healthy controls were not matched to the patients included in our analysis by clinical features that can modulate immune activity such as age or gender. As such while the findings are hypothesis generating we cannot conclude that preserved expression of these genes in a patient’s whole blood mRNA reflects the ability of a patient’s melanoma to escape immune destruction by tremelimumab.

Another limitation of the study is that patients treated in the discovery cohort were treatment naïve while patients treated in the validation cohort received prior treatment with chemotherapy. Prior chemotherapy exposure could potentially alter subsequent responsiveness to tremelimumab, the expression level of genes evaluated, and the degree of predictiveness that the gene signature identified. As noted above, the treatment-naïve discovery population had longer median and greater one-year survival than the chemotherapy-refractory validation population. The sicker validation patient population might explain why many promising biomarkers (e.g., pre-treatment CTLA4 gene expression) were up-regulated in responders versus non-responders to a statistically significant degree in the phase III but not the phase II trial. If both the discovery and validation datasets were treatment-naïve or not refractory to cytotoxic chemotherapy, additional response-predictive biomarkers would likely be identified. The treatment responses seen in both studies were mostly partial. In our analysis patients were identified as being a responder or a nonresponder. While the gene signature identified predicts for response we cannot conclude that it predicts for the degree of response.

The gene signature reported here is predictive and prognostic in the context of CTLA-4 inhibition with tremelimumab specifically. A limitation of the clinical application of the identified gene signature is that tremelimumab is not an FDA approved treatment for melanoma. Rather the CTLA-4 inhibitor ipilimumab is approved and while both antibodies inhibit CTLA-4 they are not necessarily equivalent in mechanism of action and predictive gene signatures may differ. Our analysis included only patients with both pre- and post- treatment blood samples. While this includes the majority of patients enrolled in the discovery and validation studies a limitation of our analysis is that not all patients treated in both studies were included. However a gene signature was identified and validated with this subset of patients and the discovery in the blood of a predictive gene signature provides a proof of concept. A similar strategy can be used to identify gene signatures in the context of treatment with other immune modulators including ipilimumab and anti-PD1 inhibitors.

Currently, few melanoma patients are treated with cytotoxic chemotherapy before receiving CTLA-4 and PD-1 inhibitors. Patients whose melanoma expresses V600 mutated BRAF may receive therapy with BRAF and MEK inhibitors. Often, single agent PD-1 inhibitor or combination ipilimumab plus nivolumab therapy is chosen first line. Phase III comparison of 834 stage IV melanoma patients randomized to initial treatment with pembrolizumab versus ipilimumab demonstrated significant improvement in response rate and 12 month survival, favoring PD-1 inhibitor treatment [7]. The effects of prior BRAF/MEK inhibitor, or PD-1 blockade therapy on the gene signature robustness is unknown. Presence of a transcriptional signature termed IPRES (innate anti-PD-1 resistance) in melanoma biopsy specimens was enriched for patients resistant to PD-1 inhibition but did not predict response to anti-CTLA-4 therapy [25]. Clinical trials using blood-based mRNA classifier models are warranted for PD-1 inhibitors and combinations of CTLA-4 plus PD-1 inhibitors, as their distinct targets and clinical efficacy rates differ.

This is the first large clinical study that has been validated independently in a second large clinical study to show that a response-predictive mRNA signature can be documented in blood prior to tremelimumab treatment. The signature demonstrates the potential of pre-treatment mRNA expression profiles derived from blood to predict clinical benefit. Predictive model components regulate immune activity, cell cycle proliferation, and extracellular matrix composition. Using such models can help to optimize the efficacy and safety of therapy for patients with advanced melanoma. Moreover, this strategy can be applied to PD-1 blockade and to other third generation immunomodulatory treatments currently in development.

## Conclusions

Our study demonstrates that pretreatment expression of a signature of 15 genes derived from whole blood samples obtained from patients with stage IV melanoma can predict for response to a CTLA-4 inhibitor. This allows for a minimally invasive way to identify prospectively responders to treatment. This information would optimize treatment planning allowing patients unlikely to respond to receive alternative treatments sooner, minimize toxicity risk, and allow for more efficient utilization of health care spending. Future research will need to utilize a similar approach to identify blood-based biomarkers predictive pretreatment of efficacy to anti-PD-1 based and other checkpoint inhibitor combination therapies.

## Additional file

Additional file 1: List of Genes with Full Name and Aliases of Each. (DOCX 33 kb)

## Abbreviations

AUC: Area under the curve
Ct: Cycle threshold
CTLA-4: Cytotoxic T-lymphocyte associated protein 4
FDA: Food and drug administration
IHC: Immunohistochemistry
N: Number
NPV: Negative predictive value
PCR: Polymerase chain reaction
PD-1: Programmed death-1
PD-L1: Programmed death-ligand 1
RNA: Ribonucleic acid
RRNA: Ribosomal ribonucleic acid
